# The progression of TRAb-positive subacute thyroiditis and its differential diagnosis from Graves’ disease

**DOI:** 10.3389/fimmu.2025.1727240

**Published:** 2025-12-19

**Authors:** Saiya Yuan, Xiaolu Zhao, Fan Liu, Jinming Yao, Junyu Zhao

**Affiliations:** 1 Department of Endocrinology and Metabology, The First Affiliated Hospital of Shandong First Medical University & Shandong Provincial Qianfoshan Hospital, Shandong First Medical University, Shandong Key Laboratory of Rheumatic Disease and Translational Medicine, Shandong Institute of Nephrology, Jinan, China; 2 School of Clinical Medicine, Shandong Second Medical University, Weifang, China

**Keywords:** autoimmunity, differential diagnosis, Graves’ disease, subacute thyroiditis, thyroid-stimulating hormone receptor antibody, viral triggers

## Abstract

Subacute thyroiditis (SAT) is a self-limiting thyroiditis with an unclear pathogenesis. Human leukocyte antigen (HLA)-B*35 is considered a predisposing factor, and viral infection is believed to be a trigger. In recent years, elevated levels of thyroid-stimulating hormone receptor antibodies (TRAb) have been observed in some patients with SAT. Typically, these elevated TRAb levels may spontaneously revert to negative as the disease improves. The mechanism of TRAb production may be related to abnormalities in immune surveillance and viral infections. Critically, the presence of TRAb can influence clinical manifestations, complicate diagnosis, and affect recovery in SAT. There are three types of TRAb, yet studies on their changing patterns during SAT remain scarce. Both SAT and Graves’ disease (GD) can cause thyrotoxicosis, leading to difficulties in clinical differential diagnosis, especially when SAT presents with positive TRAb. Currently, studies on TRAb-positive SAT are relatively limited. Therefore, this article reviews the pathogenesis of TRAb-positive SAT and its differential diagnosis from GD, aiming to enhance the understanding of this disease among clinicians.

## 1 Introduction

Subacute thyroiditis (SAT) is a common, self-limiting thyroid disorder, accounting for about 5% of all thyroid disorders (1). It occurs more often in women, especially between the ages of 30 and 50 (2). An epidemiological study from Olmsted County, Minnesota (2003) estimated its overall incidence at 4.9 cases per 100,000 person-years (3). Typical clinical manifestations include fever, neck pain, thyroid goiter, and elevated erythrocyte sedimentation rate (ESR) and C-reactive protein (CRP). However, during the coronavirus disease 2019 (COVID-19) pandemic, painless SAT gained attention, and neck pain is no longer considered an essential clinical manifestation (4). These nonspecific symptoms often lead to missed or incorrect diagnoses, affecting patients’ quality of life.

In recent years, the detection of high-titer thyroid-stimulating hormone receptor antibodies (TRAb) in patients with SAT has gained clinical attention (5, 6), presenting a diagnostic challenge in distinguishing it from Graves’ disease (GD). While existing reviews have summarized general SAT management (7) and some reports note TRAb in SAT cases (8, 9), a dedicated synthesis on the pathogenesis, clinical impact, and diagnostic strategy for “TRAb-positive SAT” is still lacking. To address this, this review systematically examines the mechanisms of TRAb production in SAT, evaluates its effect on disease course and prognosis, and proposes a stepwise diagnostic framework to differentiate TRAb-positive SAT from GD, aiming to offer practical guidance for clinical practice.

Note that other autoimmune thyroid disorders, such as Hashimoto’s thyroiditis, are mainly associated with thyroid peroxidase antibodies (TPOAb) and thyroglobulin antibodies (TgAb), and follow a different clinical course from SAT. Thus, these conditions fall outside the scope of this detailed discussion.

## 2 The pathogenesis of TRAb-positive SAT

The pathogenesis of SAT remains unclear. Human leukocyte antigen (HLA)-B*35 is a key susceptibility factor, first reported by Nyulassy et al. in 1975 (10) and subsequently confirmed across diverse populations (11–14). Later studies also identified associations with HLA-B*18:01, -DRB1*01, and -C*04:01 (15). Beyond genetics, viral infection is a common trigger (15). The rise in SAT cases following COVID-19 suggests severe acute respiratory syndrome coronavirus 2 (SARS-CoV-2) may be a causative agent (16). The molecular mechanisms of SARS-CoV-2-related SAT are partly understood. After viral invasion of the thyroid, antigen-presenting cells present viral particles via major histocompatibility complex class I (MHC-I) molecules to CD8+ cytotoxic T lymphocytes (CTLs), initiating bystander killing effects (17). In contrast, SARS-CoV-2 induction of GD is less clear, though helper T cell-mediated cytotoxicity may be involved (17). A retrospective study by Korkmaz et al. (18) genotyped HLA alleles in 51 SAT patients and 190 controls, stratified by COVID-19 history. Results showed that in the COVID-19(+) group, HLA-DRB1*13:02 and HLA-DRB1*13:03 were more frequent in SAT patients. In the COVID-19 (–) group, HLA-B*35:03, HLA-DRB1*12:01, and HLA-DRB1*14:01 were elevated in SAT patients, suggesting susceptibility alleles may differ based on prior COVID-19 infection. Moreover, the haplotype of HLA-A*11-B*35-C*04 was first shown to be associated with SAT, especially after SARS-CoV-2 vaccination (19). However, a recent large-scale epidemiological study challenges existing views, refuting associations between SAT and enteroviruses (e.g., echovirus, Coxsackievirus) or other viruses including SARS-CoV-2 (20). To date, the precise etiology of SAT remains unclear, with significant controversies and research gaps.

**Figure 1:**
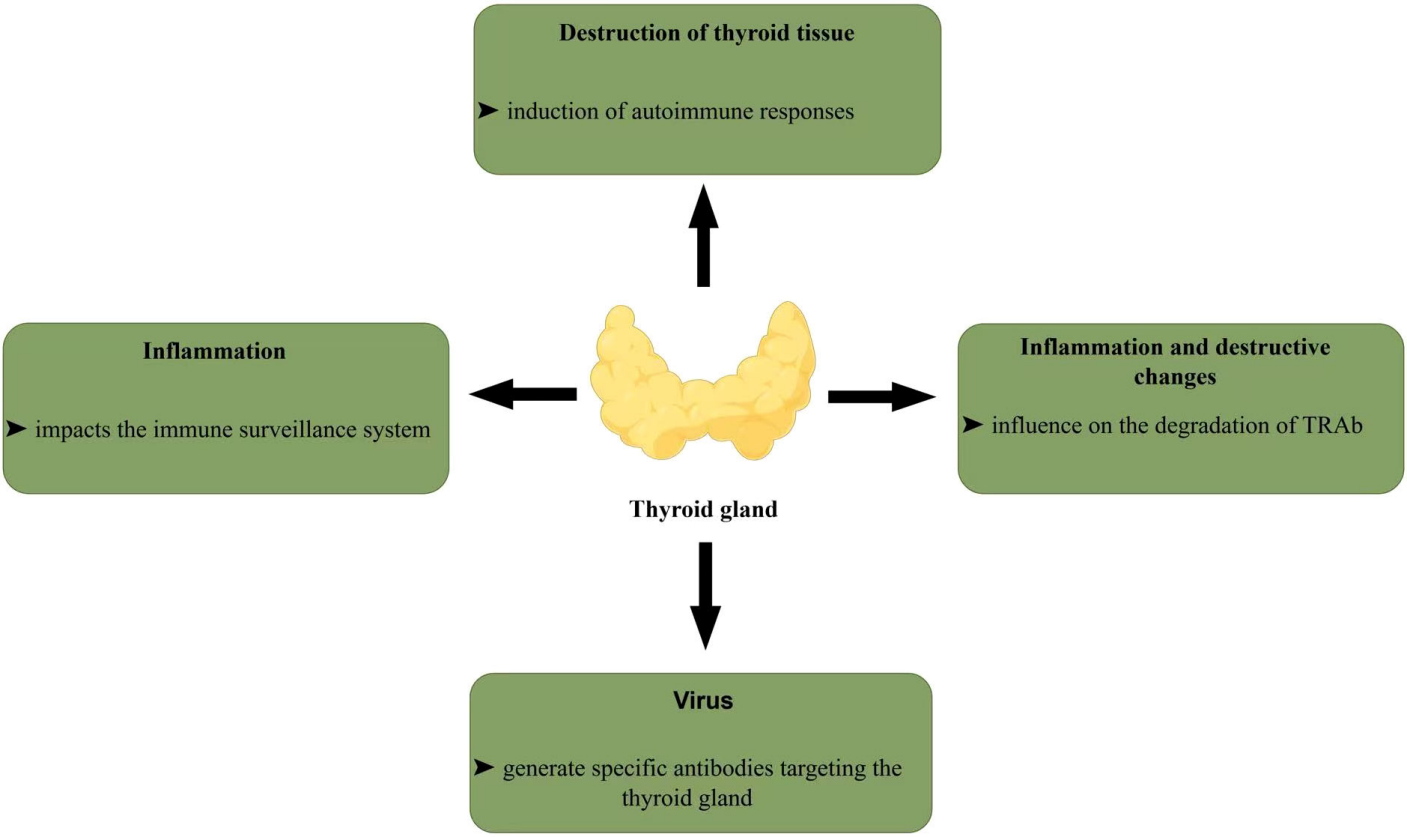
The mechanism of TRAb production in subacute thyroiditis.

In recent years, TRAb positivity has been observed during SAT. Its mechanism remains unclear but may involve the following aspects (**Figure 1**). On one hand, viral infections can damage thyroid follicular epithelial cells, potentially triggering an immune response and the production of thyroid antibodies. This may occur through molecular mimicry. Molecular mimicry refers to the phenomenon whereby self-epitopes are recognized as foreign antigens due to their structural or sequence similarity to pathogen-derived peptides (21). Evidence indicates that several peptide sequences exhibit homology between human proteins and SARS-CoV-2 viral proteins (22, 23), a similarity not observed in mammalian species unaffected by SARS-CoV-2 (22). Consequently, the adaptive immune system may generate antibodies targeting viral components, which could cross-react with structurally similar autoantigens, potentially leading to autoimmune responses (21). Several human proteins, including thyroid-derived peptides, have been identified to share sequences with SARS-CoV-2 and may thus become susceptible to cross-activation of autoreactive T and B cells by COVID-19, leading to severe autoimmunity (21). On the other hand, Sumie Fujii et al. (24) observed that thyroid tissue damage can lead to the thyroid-stimulating hormone receptor (TSHR) release into the bloodstream, where it can induce an autoimmune response to produce TRAb. Furthermore, inflammation affects the immune surveillance system, leading to autoantibody production. Inflammation and destructive changes appear to affect the degradation of TRAb. Several studies show TRAb often becomes negative as SAT resolves (24, 25), likely due to the gradual restoration of immune surveillance.

Unlike SAT, elevated TRAb titers in GD require antithyroid drug (ATD) therapy. Higher TRAb titers often necessitate longer treatment duration and higher medication doses (25). Research on the mechanism of TRAb generation in TRAb-positive SAT is still limited and needs further investigation. This research is crucial, as TRAb in SAT affects the disease’s clinical course and prognosis, and complicates differential diagnosis.

## 3 The impact of TRAb on the course and prognosis of SAT

The clinical course of SAT typically includes three phases: the thyrotoxic phase, the hypothyroid phase, and the recovery phase. The thyrotoxic phase usually lasts three to six weeks and involves widespread thyroid follicular destruction, leading to excessive thyroid hormone release. This is subsequently followed by the hypothyroid phase, which generally resolves within six months (26). However, not all patients with SAT follow this typical course. Li Chengjiang et al. (25) reported a SAT patient with a 22−month thyrotoxic phase and no transient hypothyroidism. The patient was TRAb−positive, and this positive status persisted for 22 months before resolving spontaneously. The patient’s thyroid-stimulating hormone (TSH) levels later normalized, suggesting that persistent TRAb may be linked to a prolonged thyrotoxic phase in SAT.

Regarding the prognosis of SAT, while most patients regain normal thyroid function, recurrence occurs in about 6.9-34.8% (**Table 1**). The cause of recurrence is unknown but may be HLA-dependent, linked to the coexistence of HLA-B*18:01 and HLA-B*35 (27, 28). Permanent hypothyroidism develops in approximately 6-26.8% of patients, necessitating long-term thyroid hormone replacement therapy (**Table 2**). Previous studies have found that the occurrence of persistent hypothyroidism is related to the peak TSH level within 3 months after SAT onset, but not to the levels of ESR, CRP, thyroglobulin, or the extent of thyroiditis (29–31). Other reports find no association with age, BMI, pre-treatment TSH, or initial prednisone dose, but note a correlation with cumulative prednisone dose and female sex. A cumulative prednisone dose exceeding 4 grams is the only predictor of higher hypothyroidism risk (32). Conversely, corticosteroid therapy is highly effective in preventing permanent hypothyroidism after SAT (33).

**Table 1:** Summary of recurrence rates in patients with subacute thyroiditis.

First author, year of publication	Area	Sample size (n)	Recurrence rate (%)
Hepsen, 2021	Turkey	91	17.6
Li F, 2019	China	87	6.9
Sato, 2017	Japan	42	9.5
Yotsapon, 2015	Thailand	115	12.2
Erdem, 2007	Turkey	169	12.4
T Mizukoshi, 2001	Japan	36	22.2
Bennedbaek, 1997	Denmark	23	34.8

**Table 2:** Summary of the incidence of patients with subacute thyroiditis developing permanent hypothyroidism.

First author, year of publication	Area	Sample size (n)	Incidence rate (%)
Arman Shekarian, 2024	Iran	2348	11.6
Taiba Zornitzki, 2022	Israel	38	25
Chenjia Tang, 2021	China	89	15.7
Julia Görges, 2019	Germany	127	26.8
Assim A Alfadda, 2014	Saudi Arabia	25	14.3
C A Benbassat, 2007	Israel	56	6
Vahab Fatourechi, 2003	America	160	15

Although the majority of patients with SAT are TRAb-negative, a subset of SAT patients exhibits TRAb-positivity. The TRAb status may influence disease prognosis. It is worth noting that even if there is a persistent positive antibody throughout the SAT process, it may progress to hypothyroidism. For example, Anu Alvin Mathew et al. (8) reported a SAT patient who, after prednisone treatment, developed hypothyroidism that required long-term levothyroxine therapy. TRAb remained elevated at 4.75 IU/L 16 months after onset (reference range: <2.0 IU/L), despite normal TPOAb. Persistently high TRAb levels may also serve as a predictor for subsequent GD, although this is rare. S. Al-Bacha et al. (9) identified 5 such cases among 31 patients, all male. The TRAb subtypes can transform during SAT, shifting thyroid function. Iitaka et al. (34) described a patient who initially developed thyroid-stimulating blocking antibody (TSBAb)-positive hypothyroidism, which later transitioned to thyroid-stimulating antibody (TSAb)-positive hyperthyroidism. At presentation, thyrotropin-binding inhibitory immunoglobulin (TBII) activity was primarily due to a high TSBAb titer (94%). Over the following months, both TBII and TSBAb titers rose, coinciding with a hypothyroid state. As these titers subsequently declined, the patient’s hypothyroidism resolved spontaneously. However, 18 months after the initial onset of SAT, the patient developed hyperthyroidism, now associated with elevated TSAb and undetectable TSBAb. Despite recognition of these changes, research on TRAb subtype dynamics in SAT remains limited.

## 4 Differential diagnosis of SAT and GD

SAT is an acute thyroid inflammation, typically presenting with neck pain, fever, and myalgia. In contrast, GD is an autoimmune disorder characterized by diffuse thyroid enlargement and hyperthyroidism, and may involve varying degrees of exophthalmos. GD accounts for about 85% of hyperthyroidism cases (35). The co-occurrence of SAT and GD is rare, with few cases reported (36–39). Both SAT in its thyrotoxic phase and GD can cause thyrotoxicosis with similar symptoms, making clinical differentiation difficult, especially in SAT patients without neck pain. A Japanese study found 62.1% of SAT patients were initially misdiagnosed with hyperthyroidism (40). Given their distinct causes and treatments, accurate differential diagnosis is crucial. Common diagnostic methods include TRAb measurement, radioactive iodine uptake (RAIU) testing, thyroid imaging, and thyroid ultrasonography. RAIU is considered the gold standard for distinguishing SAT from GD. However, due to its radioactivity, need for specialized equipment, and contraindications in pregnancy and lactation, it is not routinely used in clinical practice.

### 4.1 TRAb level

**Figure 2:**
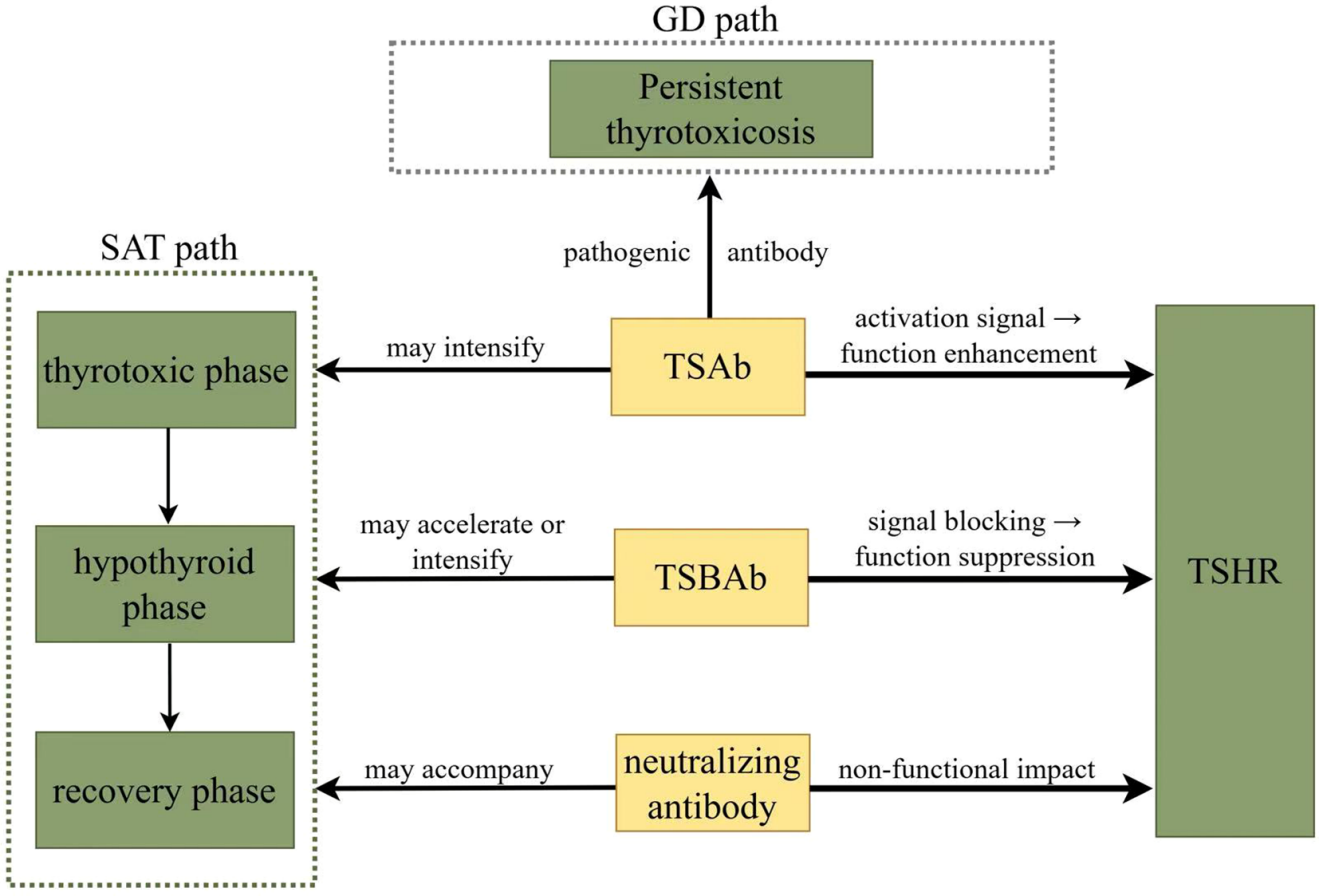
Biological effects and clinical significance of the TRAb subtype.

TRAb (also called TBII) includes three subtypes: TSAb, TSBAb, and neutralizing antibody. TSAb exerts biological effects similar to TSH but with stronger stimulatory activity. It competitively binds to TSHR, serving as a pathogenic autoantibody in GD. In contrast, TSBAb induces hypothyroidism by blocking TSH binding to its receptor. Neutralizing antibodies also bind specifically to TSHR, but their epitopes differ from those recognized by TSH, thus not interfering with TSH-TSHR interaction or subsequent biological responses (**Figure 2**). Currently, TRAb detection mainly uses receptor assays and bioassays. Receptor assays are operationally simple and rapid, including radioimmunoassay, enzyme-linked immunosorbent assay (ELISA), and chemiluminescent immunoassay. Bioassays determine subtypes by measuring cAMP levels after antibody binding. Specifically, thyroid-stimulating immunoglobulin (TSI) measures the stimulatory bioactivity of TRAb by incubating patient serum with TSHR-expressing cells and measuring cAMP production (41), with results expressed as specimen-to-reference ratio percentages (%SRR) (41). Conversely, thyroid blocking immunoglobulin (TBI) quantifies the inhibitory bioactivity of TRAb by measuring the percentage inhibition of TSH-stimulated cAMP production compared to control (41). However, bioassays are relatively cumbersome, time-consuming, costly, and technically demanding, limiting their current use primarily to research settings (42). In clinical practice, receptor assays are widely used. It is important to note that this method only indicates the presence of autoantibodies against TSHR (including TSAb and TSBAb) but does not reflect their functional status.

TRAb level testing is one of the quickest, easiest, and most economical methods for differential diagnosis. It shows high specificity for GD, with numerous studies reporting a positive rate over 95% in untreated GD patients (43). However, TRAb alone cannot fully differentiate between thyrotoxicosis caused by GD and SAT. Some GD patients with mild symptoms may have normal TRAb levels at initial presentation, especially early on (44). Others with minimal or stable thyroid volume may also test negative (45). Notably, elevated TRAb titers can occur during or after SAT (5, 6), with reported positivity rates ranging from 2.2% to 60.8% (**Table 3**). Hence, relying solely on serum TRAb positivity is insufficient to determine the cause of thyrotoxicosis.

**Table 3:** Summary of TRAb positivity in patients with subacute thyroiditis.

First author, year of publication	Area	Sample size (n)	TRAb positivity (%)
Ma Haimei, 2023	China	63	15.87
Xie Xiaoting, 2021	China	110	13.6
Julia Görges, 2020	Germany	127	2.4
Magdalena Stasiak, 2019	Polish	64	6
Ma Jianhong, 2015	China	77	60.8
Wu, Ningling, 2011	China	47	14.9
Iitaka, 1998	Japanese	1697	2.2

The commonly used clinical cutoff for TRAb in differentiating GD from SAT is 1.50 IU/L (46). However, Wang Yu et al. (46) showed that an optimal cutoff of 1.05 IU/L offers higher sensitivity and accuracy: levels above this suggest GD, while levels below are more likely SAT (normal reference range: <1.75 IU/L). Lakshmi T Naga Nitin et al. (47) found that at a TRAb threshold of 2.0 IU/L in the Indian population, diagnostic sensitivity was 97.5% and specificity 100% (normal reference range: <1.75 IU/L).

Combining multiple laboratory tests may enhance diagnostic performance compared to single tests. Ma Haimei et al. (5) found that in patients with thyrotoxicosis, an FT4/FT3 ratio ≥ 2.8 suggests the possibility of SAT, while a ratio < 2.8 points to GD. The combined detection of FT4/FT3 and TRAb demonstrated higher sensitivity and specificity in distinguishing between GD and SAT, both of which significantly exceeded those of individual tests. These results are significantly better than individual tests, indicating the potential value of promoting this combined testing method. Cai Mei et al. (48) found TPOAb and TRAb useful in distinguishing SAT from GD. Specifically, concurrent elevation of both antibodies and TRAb measured value/reference value higher than 3, may serve as distinguishing features between the thyrotoxic phase in SAT and GD.

However, most existing studies have small sample sizes and are single−center, which may introduce bias. Future large−scale, multicenter epidemiological studies are needed to validate these findings and strengthen their reliability.

### 4.2 Thyroid iodine uptake rate

RAIU measurement is another important test for identifying the cause of thyrotoxicosis. In most SAT patients, thyroid iodine uptake decreases due to follicular epithelial disruption, elevated serum thyroid hormone levels, and suppressed TSH. This creates the characteristic dissociation between high serum thyroid hormone levels and low thyroid iodine uptake. However, in some TRAb-positive SAT patients, TRAb stimulates TSH receptors on residual follicular cells, causing these remaining tissues to become hyperfunction or hypofunctional, thereby altering iodine uptake patterns.

Sumie Fujii et al. (24) reported a SAT case with high TRAb and TSAb levels and elevated RAIU, observed after CRP, ESR, FT4, and FT3 had normalized following prednisolone treatment. Shigenori Nakamura et al. (49) documented a SAT patient with both TSAb and TSBAb. During the thyrotoxic phase, despite TSH suppression, RAIU remained elevated at 35.5%. At this stage, TSAb was positive while TSBAb was negative, indicating that thyroid hormone production resulted from both destructive thyroiditis and TSAb-mediated stimulation. Three months later, when RAIU decreased to 20.7% with persistent TSH suppression, TSAb turned negative and TSBAb became positive. Five months after initial presentation, RAIU increased to 31.1%, TSH normalized, TSBAb reverted to negative, and TSAb reappeared.

These findings suggest that RAIU fluctuations in SAT may reflect the dynamic presence of TSAb. As a result, single RAIU measurements have limitations in accurately diagnosing and interpreting SAT pathophysiology.

### 4.3 Thyroid radionuclide imaging and color Doppler ultrasound

Thyroid radionuclide imaging uses technetium-99m (99mTc) to visualize the thyroid gland. It provides information on thyroid size, location, shape, structure, and both overall and regional function. The procedure involves radiation exposure and should not be repeated frequently. It is contraindicated during pregnancy and lactation, and is not useful after recent high iodine intake.

In the thyrotoxic phase of SAT, 99mTc uptake is typically low due to destruction of follicular epithelial cells, hormone release, and suppressed TSH. However, TRAb presence can lead to normal or elevated uptake in some SAT patients (25). A recent study proposed a 99mTc−pertechnetate uptake cutoff of 1.1% to distinguish GD from SAT, with 97% sensitivity and 95% specificity, but the study was limited by small sample size and single−center design. The influence of iodine intake on this test’s accuracy remains unclear (50).

Doppler ultrasound is a non−invasive method for assessing thyroid blood flow (TBF). Hisashi Ota et al. (51) demonstrated that energy Doppler ultrasound can quantify both thyroid volume and TBF, revealing TBF values exceeding 4% in all GD patients, while values in painless thyroiditis and SAT patients remained consistently below 4%. Moreover, Sajad Ahmad Malik et al. (52) reported that the peak systolic velocity of the inferior thyroid artery (PSV-ITA), measured via color Doppler ultrasound, achieved 91% sensitivity and 89% specificity in differentiating GD from thyroiditis when using an average cutoff value of 30 cm/s. These diagnostic performance metrics are comparable to those of the 99mTc-pertechnetate thyroid uptake scan. Thus, PSV−ITA measurement is particularly useful in pregnancy, lactation, after iodine exposure, or where nuclear medicine is unavailable.

Given these diagnostic options, a systematic approach can enhance clinical efficiency. We therefore propose a step-by-step integrated diagnostic algorithm to facilitate accurate and timely distinction between SAT and GD. First, evaluate clinical features and neck ultrasound for typical SAT characteristics such as pain, fever, and patchy hypoechoic areas with reduced flow. If uncertainty remains, second-line tools like the FT3/FT4 ratio and PSV-ITA can aid discrimination. As a third step, thyroid RAIU may be employed, though results should be interpreted cautiously since TRAb-positive SAT can show atypical (normal or elevated) uptake patterns. Diagnosis should integrate all clinical, sonographic, and laboratory findings rather than relying on any single test. Final confirmation comes from monitoring TRAb titer dynamics during follow-up. This protocol translates current evidence into a practical pathway to improve diagnostic accuracy.

## 5 Limitations and future directions

The current research still has the following limitations: Firstly, the generation mechanism of TRAb is still speculative and lacks direct experimental evidence; Secondly, the impact of TRAb on the long-term prognosis of SAT remains undetermined due to inconsistent data. The most crucial point is that there is currently no standardized diagnostic process that integrates these multi-dimensional parameters. Future research should prioritize large-scale longitudinal studies to clarify the natural history of TRAB-positive SAT and formulate optimized diagnosis and treatment strategies.

## 6 Conclusions

This review confirms that TRAb positive is a clinical variant type of concern in SAT, and its occurrence may be related to immune exposure after thyroid destruction and can prolong the course of thyrotoxicosis. This type of situation overlaps with the clinical manifestations of GD, making differentiation difficult. To this end, we offer a stepwise integrated diagnostic strategy to enhance the accuracy and clinical practicality of differential diagnosis.
